# The Effect of Silk Fibroin Additive on the Properties of Tannic Acid‐Based Bioadhesives

**DOI:** 10.1002/open.202500539

**Published:** 2026-02-08

**Authors:** Romina Alishiri, Reza Zeinali, Kimia Pourtaghi, Zoheir Heshmatipour, Azam Rahimi, Saeed Heidari‐Keshel, Davood Zaeifi, Farshid Sefat, Esmaeil Biazar

**Affiliations:** ^1^ Department of Biomedical Engineering To.C. Islamic Azad University Tonekabon Iran; ^2^ Group of Molecular and Industrial Biotechnology Department of Chemical Engineering Universität Politècnica de Catalunya Terrassa Spain; ^3^ Department of Microbiology To.C. Islamic Azad University Tonekabon Iran; ^4^ Department of Tissue Engineering and Applied Cell Sciences School of Advanced Technologies in Medicine Shahid Beheshti University of Medical Sciences Tehran Iran; ^5^ Hematopoietic Stem Cell Research Center Shahid Beheshti University of Medical Sciences Tehran Iran; ^6^ Proteomics research center Shahid Beheshti University of Medical Sciences Tehran Iran; ^7^ Department of Medical Biotechnology and Nanotechnology Faculty of Medicine Mashhad University of Medical Sciences (MUMS) Mashhad Iran; ^8^ Department of Biomedical Engineering School of Engineering and Digital Technology University of Bradford Bradford UK; ^9^ Interdisciplinary Research Center in Polymer Science & Technology (Polymer IRC) University of Bradford Bradford UK; ^10^ Tissue Engineering Group Department of Biomedical Engineering To.C. Islamic Azad University Tonekabon Iran

**Keywords:** cross‐linking, gelatin, polyethylene glycol, silk fibroin additive, tannic acid, tissue adhesive

## Abstract

Hydrogel adhesives have attracted significant attention for diverse biomedical applications, including tissue engineering, wound dressings, crack propagation prevention, and hemorrhage control. In this study, a tissue adhesive composed of polyethylene glycol, tannic acid, and gelatin, with different concentrations of silk fibroin (SF), was synthesized and systematically evaluated using fourier transform infrared, scanning electron microscopy (SEM), adhesion strength tests, swelling and degradation analysis, cytotoxicity and proliferation studies and antibacterial assays. Structural analysis confirmed the presence of strong hydrogen bonds among the components, while SEM imaging revealed a porous morphology for all samples. Mechanical testing demonstrated that the incorporation of SF additive significantly influenced the adhesion strength of the hydrogels. Furthermore, an increase in SF content led to a significant reduction in swelling capacity and degradation rate. The adhesive samples exhibited excellent biocompatibility, and antibacterial assays indicated that the SF additive maintained antibacterial efficacy comparable to the samples without the additive. These findings highlight the synthesized tissue adhesive as a promising candidate with favorable mechanical and biological properties for potential clinical applications in tissue engineering and wound healing.

## Graphical abstract

Hydrogel adhesive composed of polyethylene glycol (PEG), tannic acid (TA), gelatin, and silk fibroin additive, with good physical, mechanical, and biological properties, can be used in tissue engineering, wound dressing, and bleeding control.

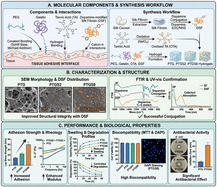

## 1 Introduction

Tissue adhesives have emerged as a transformative advancement in biomedical engineering and surgical applications, offering a viable alternative to conventional sutures and staples. Their ability to rapidly bond tissues while simultaneously promoting wound healing makes them particularly advantageous for diverse clinical scenarios, including wound closure, surgical interventions, and tissue engineering [1, 2]. Increasing interest in the development of biopolymer‐based tissue adhesives is largely driven by their intrinsic biocompatibility, biodegradability, and favorable mechanical properties, all of which are critical for ensuring patient safety and facilitating the healing process [1, 2]. Polyethylene glycol (PEG)‐based hydrogels and fibrin glue have been widely used as dural sealants to prevent cerebrospinal fluid leakage. Comparative studies suggest that PEG hydrogels may outperform fibrin glue in terms of efficacy, yet their economic impact remains uncertain in surgical procedures across Europe. A cost analysis by Talamonti et al. [3] indicated potential cost savings associated with PEG‐based hydrogels in posterior cranial fossa surgeries across five European countries. Despite their widespread use, PEG‐based sealants often exhibit inadequate mechanical strength, limiting their capacity to replace sutures fully. Shen et al. [4] developed a PEG‐based tissue patch using diacrylate F127 hydrogel and PEG diacrylate as an adhesive, demonstrating favorable tissue compatibility after 2 days of adhesion in an ex vivo pig skin model. To improve adhesion, lithium phenyl‐2,4,6‐trimethylbenzoyl phosphinate (LAP) was used as a photoinitiator, enabling rapid cross‐linking upon exposure to 395 nm UV light. Among naturally derived compounds, tannic acid (TA) has garnered significant attention due to its exceptional adhesive properties. TA, a polyphenolic compound of natural origin, has been extensively investigated for its biomedical potential, particularly as a functional component in tissue adhesives. Its unique molecular structure enables strong interactions with proteins and enhances adhesion to biological tissues. Additionally, TA exhibits antioxidant properties, which can help mitigate oxidative stress at wound sites, further contributing to its therapeutic efficacy [5, 6]. To optimize the performance of TA‐based adhesives, various additives have been explored. One promising approach involves the incorporation of aggregate particles, such as perlite, a lightweight and porous volcanic glass, which has been shown to enhance mechanical strength and overall adhesive performance [7]. The formulation of effective tissue adhesives requires a careful balance between mechanical integrity, adhesive strength, and biocompatibility to ensure functionality under physiological conditions [3]. Modifications to TA‐based adhesives have demonstrated significant improvements in adhesive performance. For instance, silver‐catalyzed polymerization of TA‐containing hydrogels has been reported to enhance stretchability and self‐adhesion [5]. Additionally, the introduction of metal ions such as Fe^3+^ has been found to increase adhesion strength to biological tissues, as demonstrated in ex vivo studies using porcine skin models [7]. The integration of dynamic cross‐linking strategies, including supramolecular interactions, has further improved mechanical resilience and reusability, making these adhesives suitable for applications requiring multiple adhesion and detachment cycles [8]. Further advancements in tissue adhesive technology include the development of ternary complex coacervates incorporating TA, PEG, and gelatin as novel bioadhesives. Li et al. [9] formulated these adhesives through simple physical blending, where adhesion strength was enhanced via programmed cross‐linking and tailored interfacial interactions between the adhesive matrix and tissue. The reinforced adhesion was attributed to the synergistic effects of TA‐mediated interfacial bonding, covalent cross‐linking between TA and gelatin for improved mechanical properties, and hydrogen‐bond‐mediated dynamic cross‐linking between TA and PEG, allowing energy dissipation under deformation. Natural biomaterials such as silk fibroin (SF) have demonstrated great potential in tissue adhesive applications. SF, a natural protein derived from *Bombyx mori* silkworms, is composed primarily of fibroin core protein (72%–81%) and sericin (19%–28%), a glue‐like coating [10, 11]. Its abundance of functional groups, including amines, carboxyl, alcohols, and thiols, facilitates multiple intermolecular interactions, rendering it a promising candidate for hydrogel‐based adhesives [12]. Recent studies have explored SF‐TA hybrid adhesives, leveraging the inherent antibacterial, antioxidant, and anti‐inflammatory properties of TA [13, 14]. The strong affinity between TA and SF is driven by hydrogen bonding, hydrophobic interactions, electrostatic forces, and van der Waals interactions, leading to the in situ formation of adhesive hydrogels [15]. In the present study, SF powders were synthesized through a simple preparation method and subsequently functionalized with dopamine hydrochloride (Dopa). This additive was incorporated at two distinct weight ratios into a PEG/gelatin/TA adhesive hydrogel to investigate its impact on key properties, including morphology, cytotoxicity, swelling behavior, degradation profile, rheological characteristics, adhesive strength, and antibacterial activity. The formulated adhesives were systematically evaluated using a series of analytical techniques to determine their suitability for biomedical applications.

## 2 Materials and Methods

### 2.1 Materials

**FIGURE 1:**
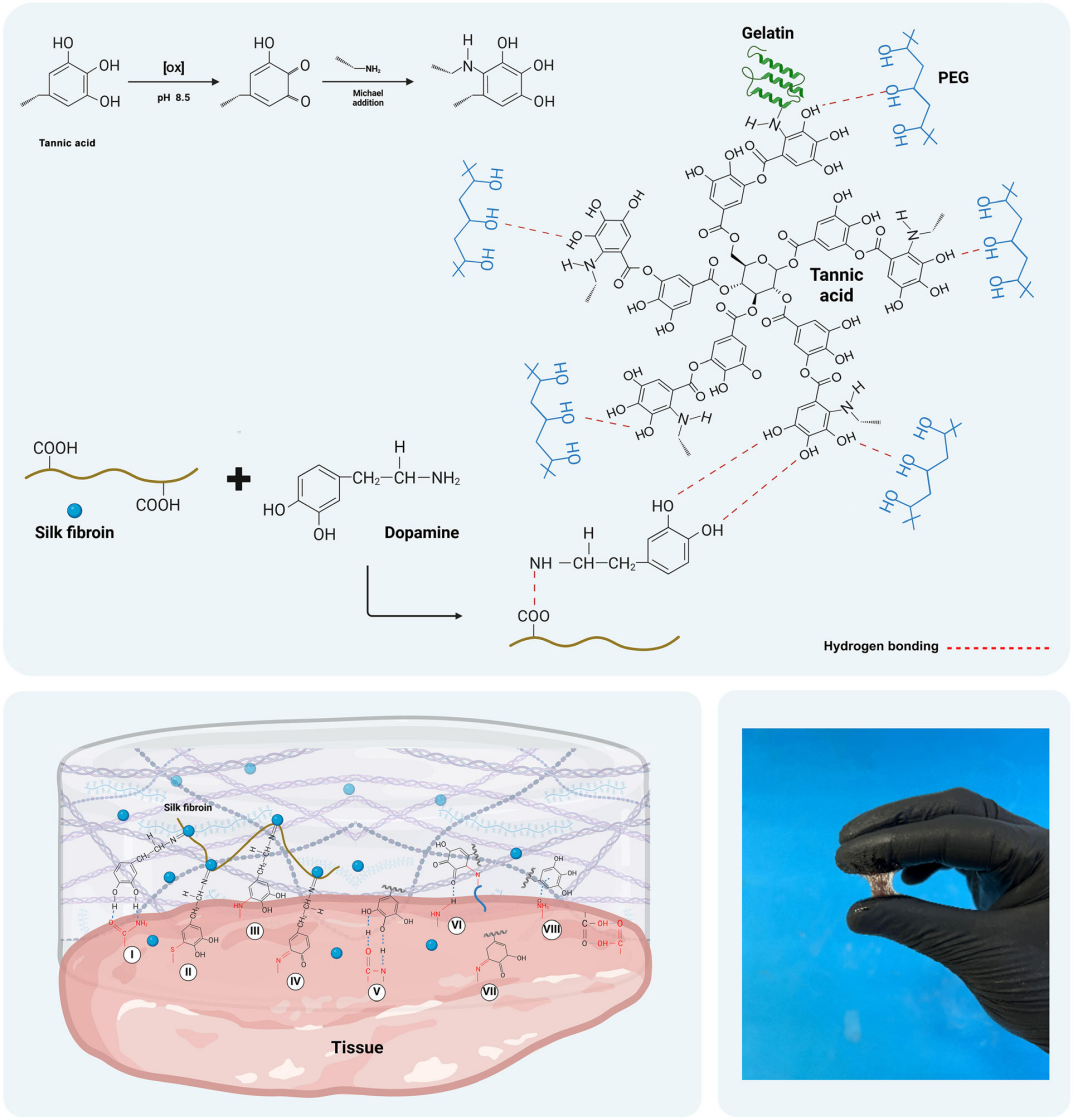
Mechanism of reaction of tissue adhesive containing PEG/gelatin, dopamine‐modified SF, and TA with tissue. Formation of covalent bonds between the catechol groups of dopamine‐modified SF (including oxidized o‐quinone moieties) with the nucleophiles present in the tissue (amines (I, III, IV) and thiol groups (II)) as well as the functional groups of TA through hydrogen bond formation (V), covalent bond (VI, VII including Michael addition and Schiff base reaction), and cation‐*π* interaction (VIII) with tissue under wet conditions.

PEG (Mw 20 kDa), *N*‐(3‐dimethylaminopropyl)‐*N*’‐ethylcarbodiimide hydrochloride (EDC), *N*‐hydroxysuccinimide (NHS), TA (ACS reagent), gelatin from porcine skin (Type A, reagent grade), lithium bromide (LiBr), Dopa, and thiazolyl blue tetrazolium (MTT) were purchased from Sigma–Aldrich (St. Louis, Missouri, USA). Fetal bovine serum (FBS), L‐glutamine, and penicillin/streptomycin were prepared by Gibco Co. (Gibco, USA). The reaction mechanisms between PEG, gelatin, and SF with TA, leading to the formation of the tissue adhesive, are illustrated in Figure 1.

### 2.2 Synthesis of Tissue Adhesive

#### 2.2.1 Silk Fibroin Extraction and Functionalization

Raw silk was degummed by boiling in a sodium carbonate (Na_2_CO_3_) solution to remove sericin proteins. The resulting fibers were thoroughly washed with distilled water and sequentially dissolved in 9.3 M lithium bromide (LiBr) at 60°C for 4.5 h. The fibroin solution was dialyzed against deionized water for 72 h using a 12 kDa dialysis membrane, then centrifuged at 4000 rpm for 10 min, stored at 4°C, and freeze‐dried for further use [12]. To enhance adhesive properties, Dopa was conjugated to SF via carbodiimide chemistry using EDC and NHS [16]. Fibroin powders at concentrations of 0.2 and 0.8 g were suspended in an ethanol/water (90:10) mixture (1 mL), with an NHS‐to‐EDC molar ratio of 2:1 (pH: 5.5) and dopamine content of 5 wt%. The suspension was then centrifuged and stored in the dark at 4°C for 48 h.

#### 2.2.2 Preparation of Tissue Adhesive Hydrogel

The oxidation of TA was carried out following a reported procedure, with little variation [17]. TA was solubilized in distilled water at a concentration of 0.02 g/mL; at this point, the temperature was increased to 60°C and 5 M NaOH was added until pH reached 9–10. Finally, an aqueous solution of H_2_O_2_ (35 wt%) was added to reach final concentration of hydrogen peroxide equal to 0.4 wt%. The mixture was allowed to react for 30 min at 60°C, and neutralized by adding 1 M HCl and oxidized TA powder was obtained after freeze drying for 3 days with a freeze dryer, at a collector temperature of −80°C. A certain amount of gelatin (10% by weight) was added to the oxidized TA, and then PEG (with a TA/PEG ratio of 3:1) was also added to the solution [9]. The dopamine‐modified SF composition was added to the above solution at specified ratios. All components were prepared separately, then combined and mixed thoroughly. The resulting mixture was sealed with parafilm and refrigerated for 24 h. The adhesive precursor was then centrifuged at 15,000 rpm for 30 min at −5°C. The supernatant was discarded, and the remaining material was poured onto a plate, forming a black sticky hydrogel. Finally, the synthesized adhesive was sterilized using ethanol and UV light.

### 2.3 Characterization

#### 2.3.1 Structural Analysis

The chemical structure of the tissue adhesive samples was analyzed using an fourier transform infrared (FTIR) spectrometer (4700, JASCO, Japan). Dried samples were finely ground with potassium bromide (KBr) to form a homogeneous mixture, which was then compressed into pellets. Spectra were recorded at room temperature (25°C) over a scanning range of 500–4000 cm^−1^. Spectroscopic technique (UV–vis, Hitachi U‐3900) was used to investigate the conjugation of dopamine with SF.

#### 2.3.2 Morphological Analysis

To examine the morphology of the SF powder and tissue adhesive (with and without SF), samples were freeze‐dried and analyzed using scanning electron microscopy (SEM) (XL30 Philips, Eindhoven, The Netherlands). Prior to imaging, samples were coated with a thin layer of gold, and SEM imaging was performed at an accelerating voltage of 20 kV.

#### 2.3.3 Mechanical Test

The adhesive strength of the PEG/TA/gelatin samples (PTG) containing 0.2 and 0.8 g of SF, designated, respectively, as PTGS2 and PTGS8, was evaluated using 0.5 g of gel per variant. Adhesion strength was assessed by applying weights of 100 g, with measurements repeated three times for statistical reliability. A universal testing machine (SANTAM STM‐20) was used to evaluate the adhesion strength of adhesives to bovine skin in accordance with the standard (ASTM F2255−2015). The lap‐shear test was conducted to obtain the shear strength under adhesive curing conditions for 24 h at 37°C and testing in phosphate‐buffered saline (PBS) solution with pH 7.4. Rheological properties of both modified and unmodified hydrogels were analyzed using a rotational rheometer (Physica MCR300, USA) within a frequency range of 1–100 Hz and an amplitude of 1% at 25°C. The data included viscosity, elastic modulus, and viscous modulus.

#### 2.3.4 Swelling and Degradation Analysis

For swelling analysis, dried samples were weighed (*W*_d_) and immersed in water for 2 h. After surface blotting with filter paper, samples were reweighed (*W*_w_) and monitored over time without drying. The swelling ratio was calculated using the Equation (1)

(1) $$\mathrm{Swelling}\;\mathrm{ratio}\;(\%)\;=\;[(W_{w}-W_{d})/W_{d}]\;\times \;100\;$$

Degradation analysis was conducted by cutting thin, compact samples, weighing them in their dry state (*W*_i_), and immersing them in PBS at 37°C under continuous shaking (50 rpm). At designated time points, samples were oven‐dried and reweighed (*W*_f_). The percentage weight loss was determined using the Equation (2)

(2) $$\mathrm{Weight}\;\mathrm{loss}\;(\%)\;=\;[(W_{i}-W_{f})/W_{i}]\;\times \;100$$

### 2.4 In Vitro Analysis

#### 2.4.1 Cytotoxicity Assay

The biocompatibility of the adhesives was evaluated using the MTT assay by assessing epithelial cell viability and proliferation in comparison to standard 2D culture conditions. Human corneal epithelial cells, isolated following a previously established protocol [11], were cultured in Dulbecco's Modified Eagle Medium/Nutrient Mixture F‐12 (DMEM/F‐12) supplemented with 10% FBS, 2 mM L‐glutamine, and 100 µg/mL penicillin‐streptomycin (Gibco, USA). Sterilized adhesive samples were cut to size, placed in 96‐well plates, and preincubated in culture medium for 24 h. Subsequently, 1,000 cells per well were seeded and incubated at 37°C with 5% CO_2_ for 24, 48, and 72 h. Following incubation, the culture medium was replaced with 100 µL of MTT (Sigma, Germany) solution, and the samples were incubated for an additional 3–4 h. Formazan crystals were then dissolved, and absorbance was measured at 570 nm using a spectrophotometer.

#### 2.4.2 DAPI Staining Assay

After sterilization, adhesive samples were cut into small pieces, seeded with 2,000 cells per well in a 48‐well plate, and incubated for 72 h. The culture medium was then removed, and the samples were stained with DAPI (Sigma, Germany) for 15 min. Fluorescence microscopy was used to visualize cell infiltration. The adhesive samples displayed cream‐to‐brown halos, which affected dye absorption and altered fluorescence intensity due to variations in color and thickness.

#### 2.4.3 Microbial Analysis

The antibacterial activity of the adhesives was evaluated using a well‐plate diffusion method against *Escherichia coli*, *Pseudomonas aeruginosa*, and *Staphylococcus aureus*. Bacterial suspensions were standardized to ~3 × 10^8^ CFU/mL using the McFarland method. Wells were created on agar plates, and 100 µL of sterile medium was added to each well. Subsequently, 0.05 g of the adhesive, along with control and blank samples, were placed into the wells. After a 24 h incubation, clear zones of inhibition were measured, indicating antibacterial activity. All experiments were conducted in triplicate, and results were reported as mean ± standard deviation. Statistical significance was determined using Student's *t‐*test and two‐way analysis of variance with Tukey's post hoc test (*p* < 0.05 within groups; *p* < 0.01 between groups).

## 3 Results and Discussion

**FIGURE 2:**
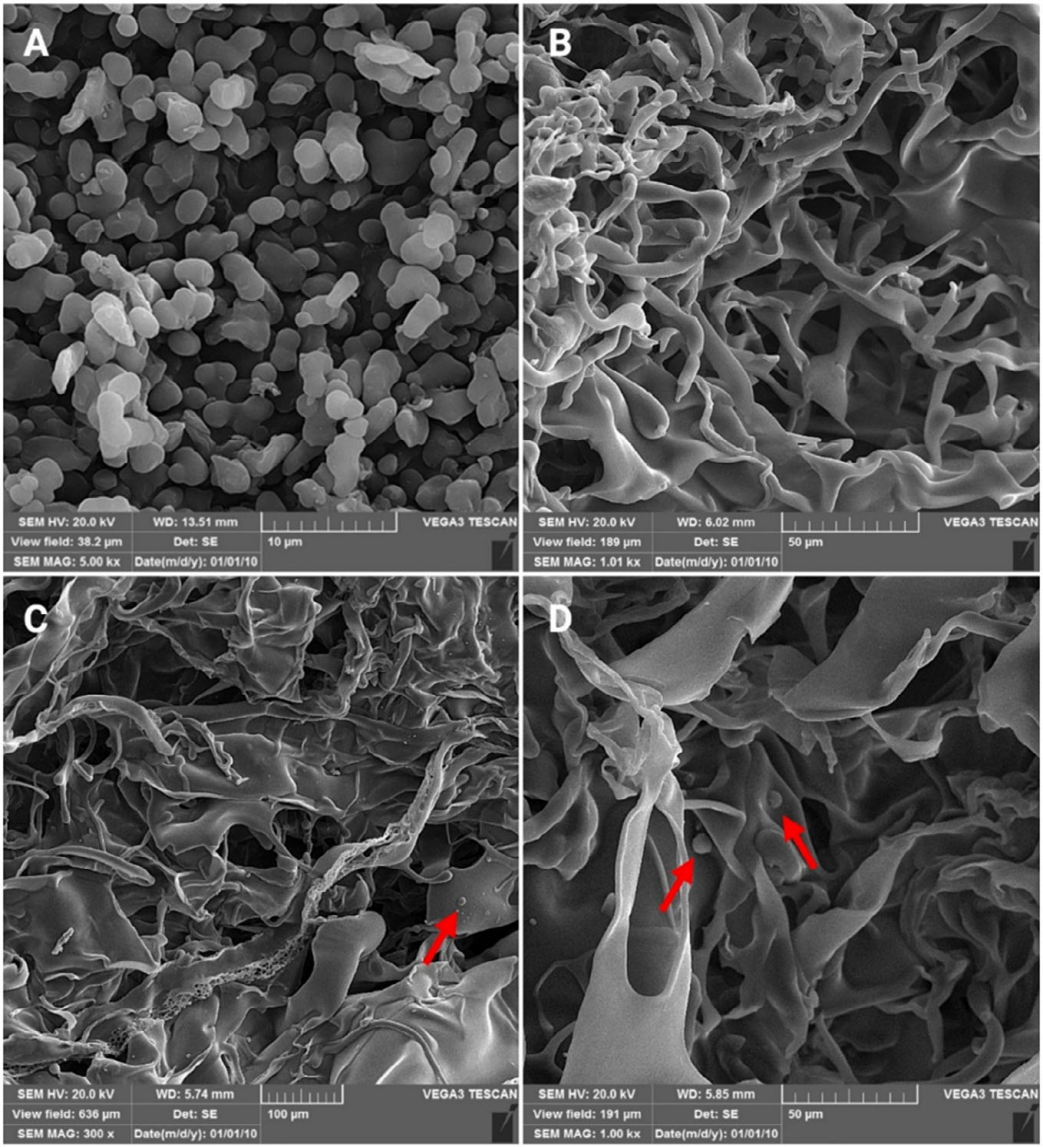
SEM images of the samples: (A) SF powders, (B) PTG, (C) PTGS2, and (D) PTGS8 (Arrows: SF powders).

The SEM image in Figure 2A shows that the SF particles exhibit high homogeneity, with an average particle size of ≈2 µm, confirming the effectiveness of the extraction method. Figure 2B presents the morphology of the PTG sample (PEG/TA/gelatin) without additives, displaying a well‐defined porous structure with interconnected pores. The addition of SF (Figure 2C,D) did not significantly alter the structural morphology compared to the PTG sample. The arrows in the figures indicate the uniform distribution of SF powders within the matrix.

**FIGURE 3:**
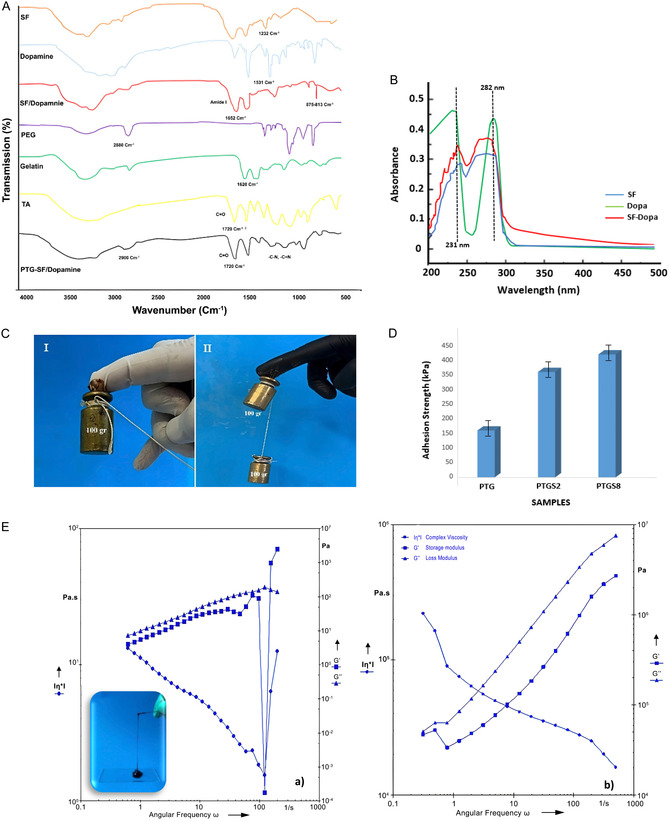
(A) FTIR spectra of the samples, (B) UV–vis spectra of SF and SF‐Dopa, (C) bonding performances of adhesives (I: PTG; II: PTGS8) as applied on weights, (D) adhesion strength of various TPG formulations measured using Lap‐shear test, and (E) rheological diagrams of the adhesives (a: PTG; b: PTGS8).

FTIR spectra (Figure 3A) confirmed the presence of SF in the adhesive matrix. The characteristic amide I peak at 1652 cm^−1^ and amide II peak at 1531 cm^−1^ (N—H bending vibration) indicate the protein structure. The amide III peak at 1232 cm^−1^ confirms the presence of SF. Additionally, the SF‐Dopa spectrum displayed peaks at 875 and 813 cm^−1^, corresponding to ortho‐dihydroxy groups and dopamine functionality, respectively. The gelatin spectrum exhibited an O—H stretching band around 3350 cm^−1^, a weaker CH_2_ peak at 2922 cm^−1^, and amide I and II/III vibrations at 1540 and 1240 cm^−1^. The PEG spectrum showed O—H stretching between 3550 and 3200 cm^−1^, C—H stretching at 2840–3000 cm^−1^, and C=O stretching between 1750 and 1735 cm^−1^. The TA spectrum displayed a prominent C=O band at 1720 cm^−1^. Notably, in the PEG/TA/gelatin formulation, the C—H stretching band shifted from 2880 to 3000 cm^−1^, and the C—N/C=N stretching bands decreased, indicating covalent bonding between TA and the —NH_2_ groups of gelatin. A reduction in O—H stretching intensity suggests interactions between TA, gelatin, PEG, and SF. As shown in Figure 3B, compared with SF, the sample of SF‐Dopa under UV–vis spectroscopic analysis showed enhanced absorption near the wavenumbers of 231 and 282 nm; this demonstrates the successful incorporation of SF with dopamine as well [18]. The lack of absorption around 395 nm revealed that the catechol groups were not oxidized to quinones during the reaction process [19]. All these changes denote that dopamine was successfully conjugated with SF. Adhesion strength tests (Figure 3C) demonstrated that the PTG sample maintained adhesion stability under a 100 g weight for 5 s. The addition of SF significantly enhanced adhesion, with the PTGS8 sample sustaining two 100 g weights for 15 s, whereas the PTGS2 sample failed after 10 s. These results suggest that higher concentrations of SF improve the adhesive performance of the formulation. The results of lap‐shear test showed that the adhesion strength of PTGS8 reaches a maximum of around 420 kPa, while that of PTGS2 is about 360 kPa, which is more than two times that of the sample without additives (160 kPa) (Figure 3D). Rheological analysis revealed notable differences in viscosity, elastic modulus, and viscous modulus with the addition of SF (Figure 3E). The control sample (PTG) exhibited a viscous modulus and elastic modulus of ≈100 Pa at 10 Hz, with a complex viscosity of about 10 Pa.s. With the addition of SF, all three parameters increased significantly, with PTGS8 showing an elastic modulus of 6 × 10^5^ Pa and a viscous modulus of 4 × 10^5^ Pa at 10 Hz. The complex viscosity increased to ~4 × 10^4^ Pa.s, indicating the formation of intramolecular interactions and physical trapping effects that restrict polymer chain movement.

**FIGURE 4:**
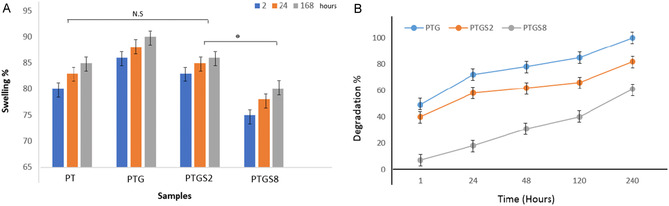
(A) Swelling amount and (B) degradation percentage of tissue adhesive samples at different times.

Swelling studies (Figure 4A) indicated that the PEG/TA sample exhibited an 85% swelling rate after 168 h. The addition of gelatin increased swelling to 90%, attributed to gelatin's hydrophilic nature. However, SF reduced swelling, with PTGS2 and PTGS8 exhibiting swelling rates of 87% and 77%, respectively. Degradation analysis (Figure 4B) showed that the PEG/TA/gelatin sample completely degraded within 10 days. The inclusion of SF decreased degradation, with PTGS2 and PTGS8 showing 82% and 61% degradation, respectively, after 10 days. These results suggest that SF enhances the structural integrity and stability of the adhesive formulation.

**FIGURE 5:**
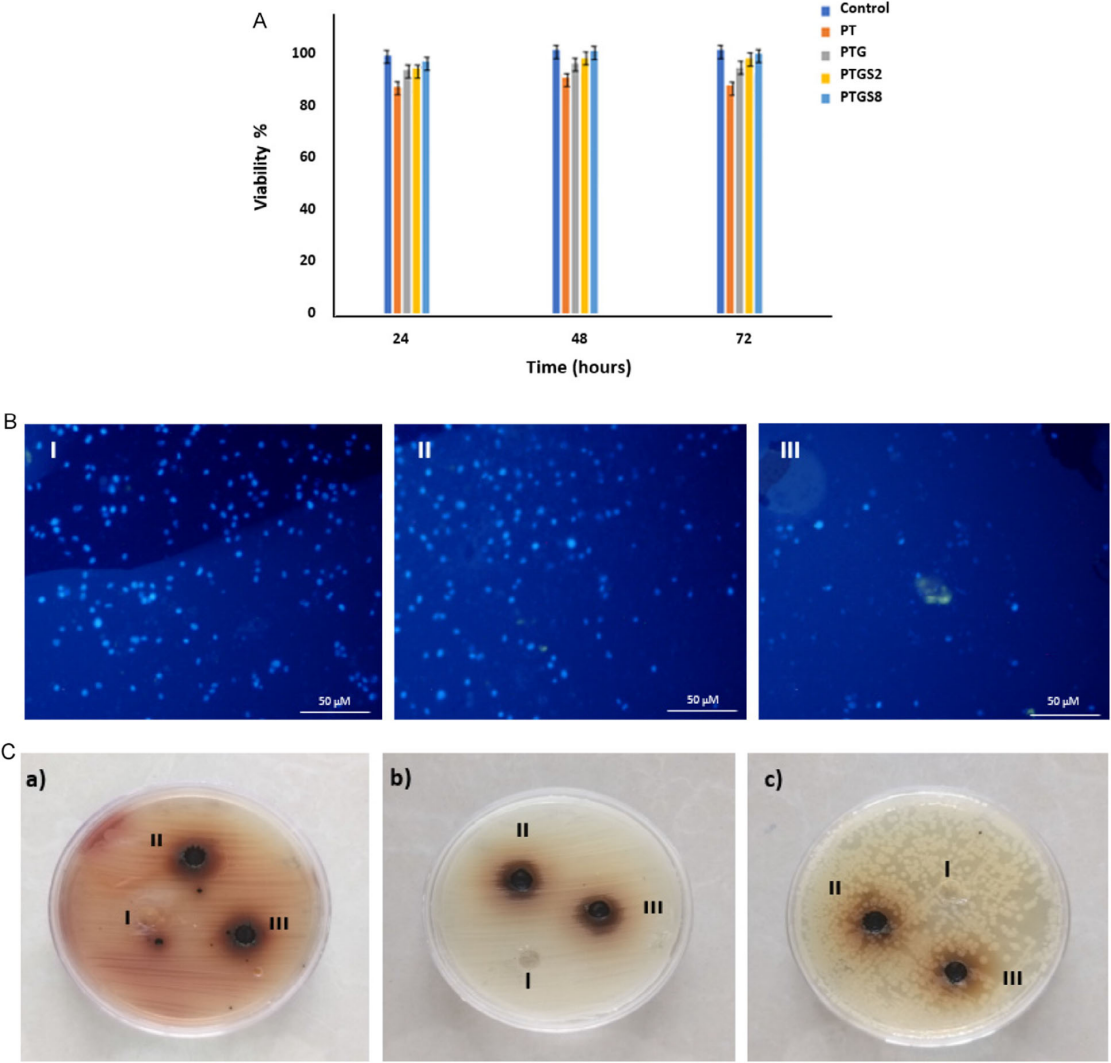
(A) Toxicity assay of adhesive samples at different time points. Control: tissue culture polystyrene. (B) Microscopic images of DAPI‐stained tissue adhesive samples: (I) PTG sample, (II) PTGS2 sample, and (III) PTGS8 sample, and (C) microbial analysis of tissue adhesive samples (I: Control [PEG/gelatin], II: PTG, III: PTGS8) against different bacteria (a: *P. aeruginosa*, b: *S. aureus*, c: *E. coli*).

Cell viability analysis (Figure 5A) demonstrated that samples containing SF supported cell proliferation comparable to the control. However, samples lacking gelatin and cross‐linked with TA exhibited slight cytotoxicity, likely due to TA release. This effect was mitigated by the addition of gelatin and SF, underscoring their role in enhancing biocompatibility. DAPI staining (Figure 5B) revealed well‐stained cell nuclei in the PTG sample (Figure 5BI). In contrast, the PTGS2 and PTGS8 samples (Figure 5BII,III) displayed fewer visible cells, likely due to increased cross‐linking, which reduced porosity and limited cell penetration. Antibacterial tests (Figure 5C) demonstrated distinct inhibition zones for different bacterial strains. The PTG and PTGS8 samples exhibited inhibition zones of ~15 mm against *P. aeruginosa* and *S. aureus* (Figure 5Ca,b), confirming moderate antibacterial activity. However, none of the formulations exhibited antibacterial activity against *E. coli* (Figure 5Cc). These results indicate that the adhesives are particularly effective against Gram‐positive bacteria.

PEG‐based adhesives are widely used in industrial and consumer products due to their excellent adhesion properties and relatively low toxicity. Tissue wounds pose a significant challenge to the healthcare system, affecting millions worldwide. Traditional wound closure methods such as suturing and stapling have limitations, including inadequate wound coverage, fluid leakage, and increased risk of infection. Consequently, there is a pressing need for more effective solutions adaptable to diverse wound conditions. Adhesive hydrogels present a promising alternative for wound care, offering firm sealing without leakage, ease of application, mechanical support, and flexibility. However, in vivo durability is often hindered by excessive swelling and unpredictable degradation, limiting their practical applications. While PEG‐based tissue adhesives have been widely used in surgical procedures, their weak mechanical strength prevents them from fully replacing sutures. A ternary complex coacervate consisting of TA, PEG, and gelatin (TPG) has been proposed as a novel bioadhesive, fabricated via a simple physical blending method. The adhesion capacity of TPG was enhanced by programming the cross‐linking network and tailoring interfacial interactions with tissue. Optimized formulations, particularly those incorporating 10% (m/m) gelatin (TPG10), demonstrated adhesion strengths over three times that of TPG0 (without gelatin) after 24 h of curing at pH 6°C and 37°C. The enhanced adhesion mechanism involved TA providing strong interfacial adhesion, TA‐gelatin covalent cross‐linking improving mechanical integrity, and dynamic hydrogen bonding between TA and PEG dissipating energy upon deformation. Additionally, TPG10 exhibited antibacterial activity, biocompatibility, and suitable degradation properties, demonstrating efficacy in wound closure and tissue repair in rat skin wound models [9]. Jing et al. [14] reported the synthesis of a bio‐functional hydrogel from SF and TA. Due to TA's self‐assembling properties and favorable molecular interactions with SF, this formulation resulted in a hybrid hydrogel with rapid gelation, good adhesiveness, shear‐thinning, and self‐recovery properties. In another study, SF was functionalized with dopamine through a one‐step cross‐linking process to enhance its adhesive strength. PEG was incorporated to optimize gelation time, resulting in NESFB‐Dopa, a bioadhesive with superior adhesive strength under both dry and wet conditions compared to clinically used fibrin sealants [16]. In this study, an adhesive composed of TA, PEG, and gelatin, similar to Li et al., was developed, with the addition of dopamine‐modified SF (SF‐Dopa) to enhance bioadhesive properties. The goal was to improve adhesion strength and reduce setting time while maintaining biocompatibility and antibacterial properties. SF‐Dopa mimics mussel adhesive proteins, providing excellent cell adhesion in wet environments with high biocompatibility, though its mechanical strength is lower than that of synthetic bioadhesives [16]. Our results demonstrated that SF significantly increased adhesion and rheological properties while reducing swelling and degradation rates. These effects can be attributed to the physical entrapment of polymer chains by SF particles and the lower hydrophilicity of SF compared to gelatin. TA plays a crucial role in inhibiting bacterial growth and biofilm formation, which is a key concern in wound healing and surgical applications [20, 21]. Its ability to disrupt bacterial membranes and suppress extracellular polymeric substance production highlights its antimicrobial potential in tissue adhesives [20]. This antimicrobial activity enhances the safety profile of TA‐based adhesives and improves wound healing outcomes by minimizing infection risks. Jing et al. [14] demonstrated the antibacterial activity of SF‐TA gels against both Gram‐negative (*E. coli*) and Gram‐positive (*S. aureus*) bacteria through agar diffusion assays. The SF‐TA gel exhibited inhibition zones of 9 mm for E. coli and 17 mm for *S. aureus*, suggesting inherent bacterial membrane lytic properties. Additionally, Egan et al. [22] reported that SF solutions possess inherent antimicrobial effects, with concentrations ≥4% *w*/*v* inactivating *S. aureus* and *P. aeruginosa*. However, their findings also indicated that while SF solutions inhibit bacterial survival in storage, their efficacy in simulated infected wounds diminishes in the presence of nutrients, necessitating terminal sterilization of final products. Our study demonstrated that TPGS adhesives exhibit strong antibacterial properties against *P. aeruginosa* and *S. aureus* due to the presence of TA in the coacervates. All samples showed clear inhibition zones for these bacteria, whereas no inhibition was observed for *E. coli*. This selective antibacterial activity aligns with previous reports on TA‐containing formulations [23, 24]. Importantly, the addition of SF improved the physical and mechanical properties of the tissue adhesive without compromising its antibacterial efficacy, maintaining the same level of activity as the control sample without additives. These findings highlight the potential of SF‐enhanced tissue adhesives as multifunctional bioadhesives with superior adhesion, durability, and antimicrobial properties.

## 4 Conclusions

Bioadhesives play a crucial role in surgery for hemostasis, tissue sealing, and wound healing. However, many existing bioadhesives face challenges such as weak adhesion in wet environments, inadequate sealing, and suboptimal clotting performance. This study evaluated the effects of SF as an additive in PEG‐based adhesives through comprehensive analyzes. The findings demonstrated that SF enhances key properties, particularly adhesion strength, while also regulating swelling and degradation rates, all without compromising the antibacterial efficacy of the adhesive. Overall, the incorporation of SF offers a promising strategy for improving bioadhesive performance, expanding their potential applications in wound healing, hemorrhage control, and other biomedical fields.

## Author Contributions

**Romina Alishiri**: data curation (equal), formal analysis (equal), investigation (equal), methodology (equal), project administration (lead), validation (equal), writing – original draft (equal). **Reza Zeinali**: conceptualization (equal), data curation (equal), formal analysis (equal), investigation (equal), validation (equal), writing – original draft (equal). **Kimia Pourtaghi**: data curation (equal), investigation (equal), methodology (equal), resources (equal). **Zoheir Heshmatipour**: data curation (equal), formal analysis (lead), investigation (equal), methodology (lead), validation (lead), visualization (lead). **Azam Rahimi**: data curation (equal), investigation (equal), methodology (equal), validation (equal), visualization (equal). **Saeed Heidari‐Keshel**: conceptualization (equal), data curation (equal), formal analysis (equal), methodology (lead), resources (equal), supervision (supporting), visualization (equal). **Davood Zaeifi**: formal analysis (equal), investigation (equal), software (lead), validation (equal), visualization (equal), writing – original draft (equal). **Farshid Sefat**: data curation (equal), formal analysis (supporting), investigation (equal), methodology (equal), validation (equal), visualization (equal). **Esmaeil Biazar**: conceptualization (lead), data curation (equal), formal analysis (equal), funding acquisition (lead), investigation (equal), methodology (equal), supervision (lead), validation (equal), visualization (equal), writing – review & editing (lead).

## Funding

This research received no external funding.

## Ethics Statement

Not applicable.

## Conflicts of Interest

The authors declare no conflicts of interest.

## Data Availability

The data used to support the findings of this study are available from the corresponding author upon request.
